# Author Correction: PatCID: an open-access dataset of chemical structures in patent documents

**DOI:** 10.1038/s41467-024-55566-3

**Published:** 2025-01-02

**Authors:** Lucas Morin, Valéry Weber, Gerhard Ingmar Meijer, Fisher Yu, Peter W. J. Staar

**Affiliations:** 1 IBM Research, Säumerstrasse 4, 8803 Rüschlikon, Switzerland; 2 Department of Information Technology and Electrical Engineering, ETH Zürich, Sternwartstrasse 7, 8092 Zürich, Switzerland

**Keywords:** Cheminformatics, Literature mining, Scientific data

Correction to: *Nature Communications* 10.1038/s41467-024-50779-y, published online 02 August 2024

The original version of this article contained an error in Fig. 3, in which the labels “Only Reaxys (manual)” and “Only Reaxys (automatic)” were inverted.

Additionally, the original version of the legend of Fig. 3 contained an error in the second sentence, which incorrectly read: “For the top bar, 33.5% of molecules in D2C-RND are covered with both Reaxys (manual annotations) and PatCID, 22.5% in PatCID only, 8.0% in Reaxys (manual annotations) only, and 36.0% in none of the databases.” The corrected version states “For the top bar, 33.5% of molecules in D2C-RND are covered with both Reaxys (automatic annotations) and PatCID, 22.5% in PatCID only, 8.0% in Reaxys (automatic annotations) only, and 36.0% in none of the databases.” This has been corrected in both the PDF and HTML versions of the article.

