# Supplementary material for: PatCID: an open-access dataset of chemical structures in patent documents
Source: Nat Commun. 2024 Aug 2;15:6532. doi: 10.1038/s41467-024-50779-y (PMC11297020; doi:10.1038/s41467-024-50779-y)
Supplement: Supplementary file 1 — Supplementary Information [file 41467_2024_50779_MOESM1_ESM.pdf]

Supplementary Information: PatCID: an open-access dataset of chemical structures in patent documents

Lucas Morin[1, 2] Valéry Weber[1] Gerhard Ingmar Meijer[1]
Fisher Yu[2] Peter W. J. Staar[1]

[1] IBM Research, Säumerstrasse 4, 8803 Rüschlikon, Switzerland
[2] Department of Information Technology and Electrical Engineering, ETH Zurich, Sternwartstrasse 7, 8092 Zürich, Switzerland

Supplementary Note 1

In order to select which documents are included in PatCID, the search 'alkyl' was used. 'alkyl' is a relevant search proxy for documents in the field of organic chemistry since documents displaying Markush structure images almost always contain the term 'alkyl' in an associated Markush text description. An alternative would have been to select documents associated with International Patent Classification (IPC) codes C07, C08, and C09. These codes refer to documents in the field of organic chemistry. However, by selecting only these documents, many relevant documents would have been missed. Indeed, a patent document containing molecule images can be classified with an IPC code that refer to its application domain, for instance medical science (A61) or electric elements (H01), rather than organic chemistry. The search proxy 'alkyl' was used for all patent documents, with the exception of those published after 2001 in the United States. In this case, documents with at least one MOL file annotation directly provided by the United States Patent and Trademark Office were selected.

Between 2010 and 2019, the patent offices from the United States, Europe, Japan, Korea, and China published 1.06M patent families in the field of organic chemistry, while all 107 patent offices worldwide published 1.16M. These 5 patent offices cover 90% of published patent documents in the field of organic chemistry. For the same time period, about 70% of patent families in the field of organic chemistry from Asian Pacific offices were not extended to the United States. These numbers are obtained by searching patents which publication year is between 2010 and 2019, and which contain the text 'alkyl' in LexisNexis TotalPatent One [1]. The associated query is: FT:(*alkyl*) AND PD:[2010-01-01 TO 2019-12-31].

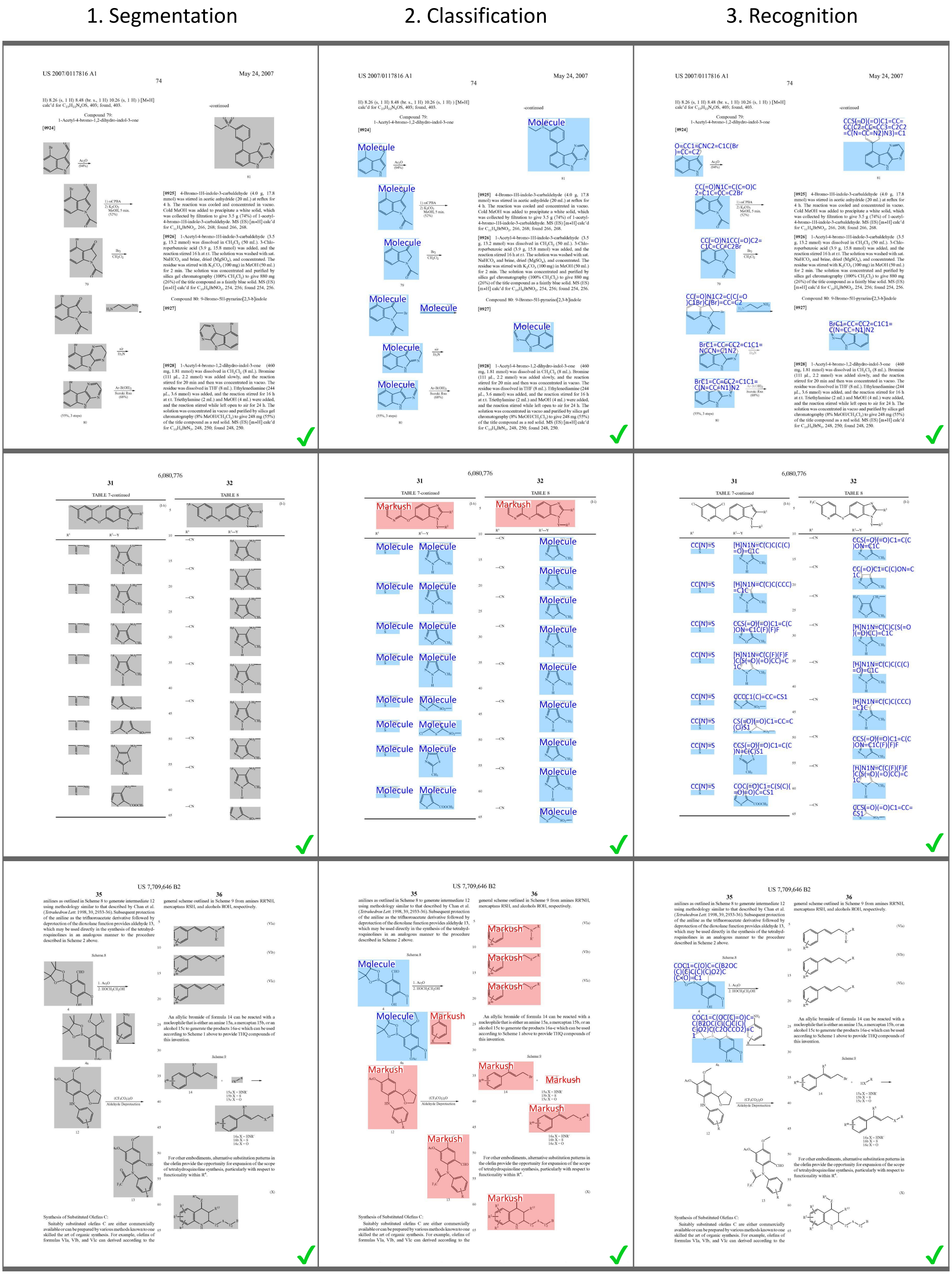

Supplementary Figure 1: **Ingestion pipeline qualitative evaluation.** Examples of correct predictions of the segmentation, classification, and recognition modules. Each row is an example page, with its segmented chemical images (in gray), its molecular structures ('Molecule' in blue), its Markush structures ('Markush' in red), and its annotated SMILES.

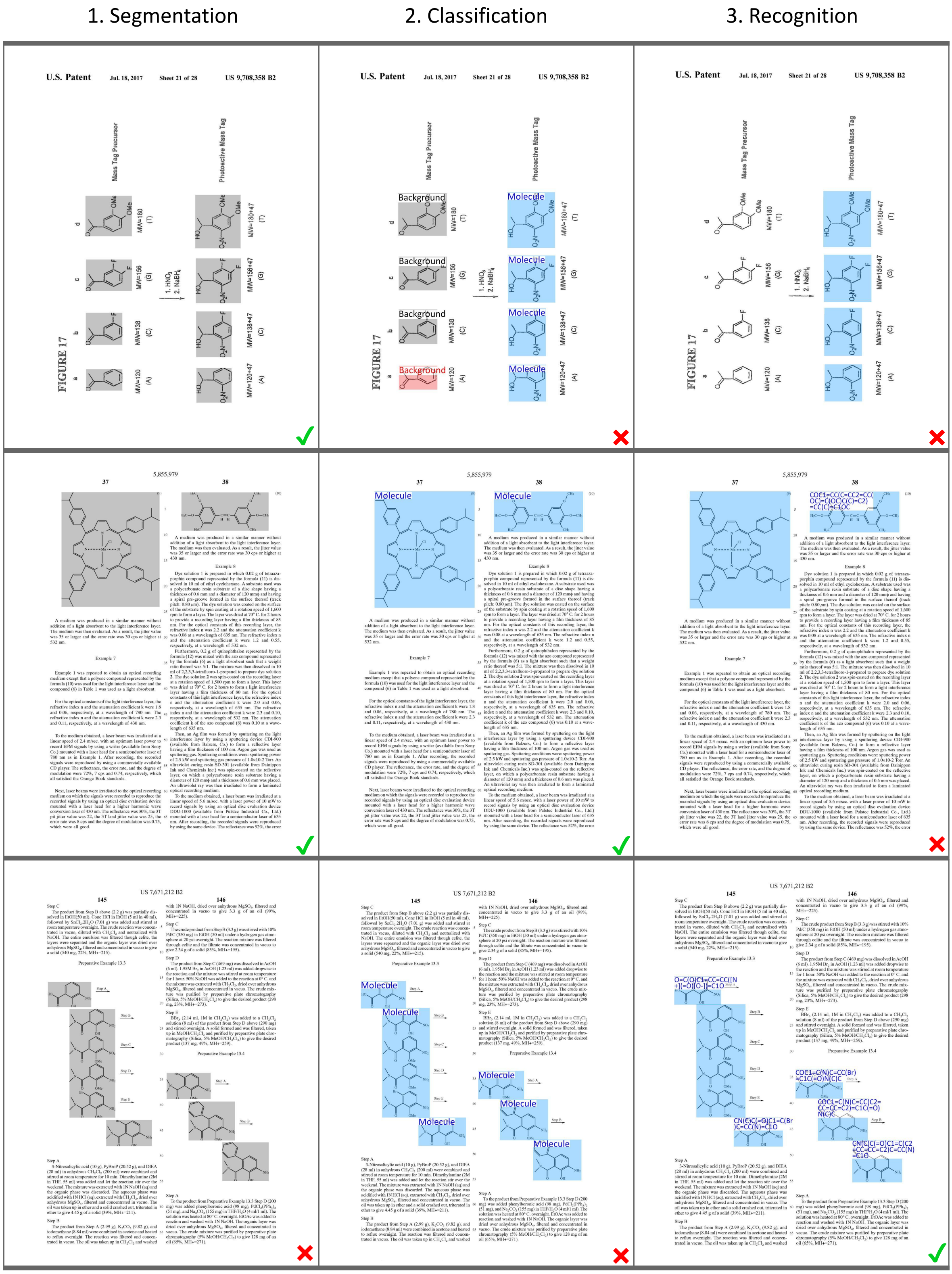

Supplementary Figure 2: **Ingestion pipeline failure cases.** Examples of incorrect predictions of the segmentation, classification, and recognition modules. Each row is an example page, with its segmented chemical images (in gray), its molecular structures ('Molecule' in blue), its Markush structures ('Markush' in red), and its annotated SMILES.

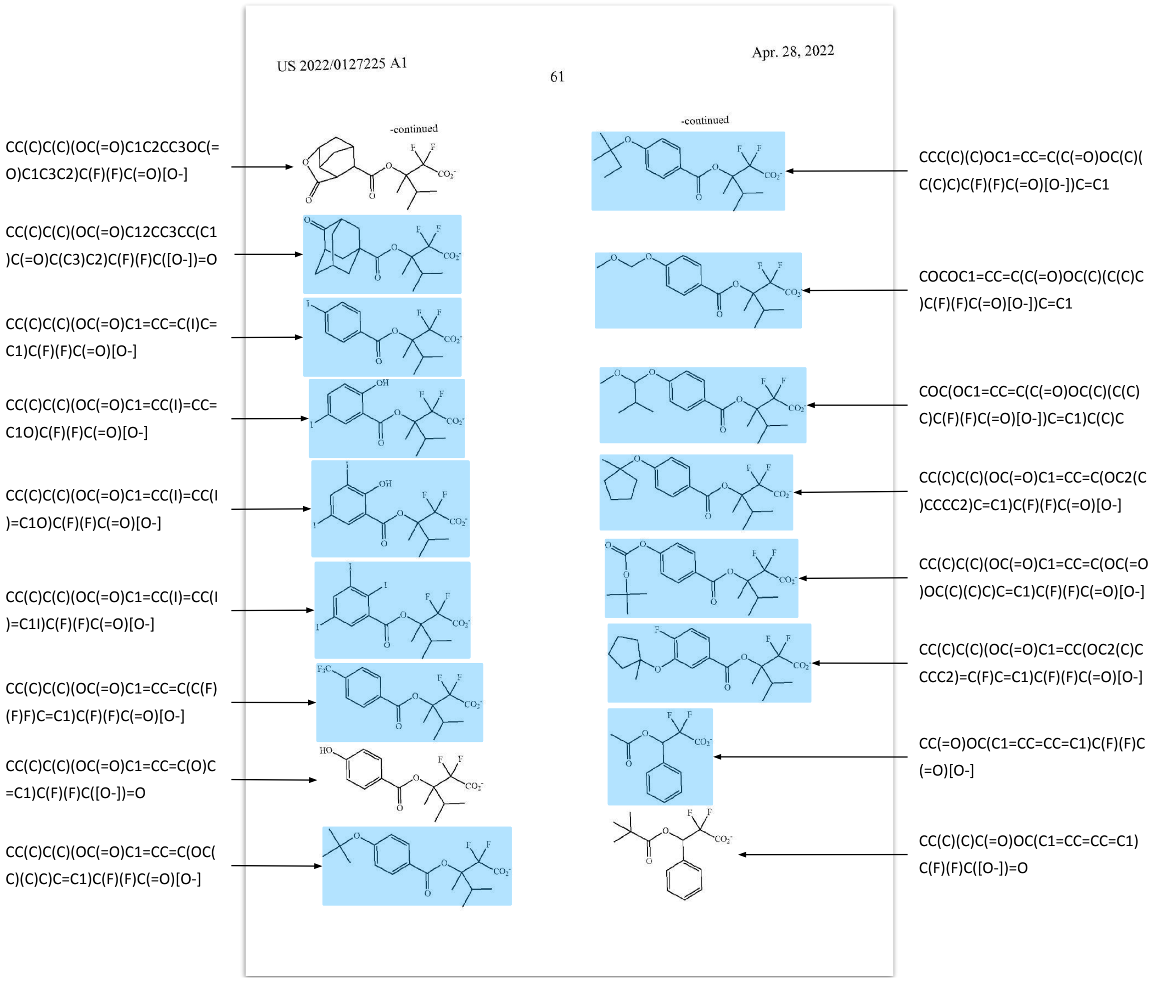

Supplementary Figure 3: **Qualitative example of PatCID document coverage advantage.** Page from US20220127225, selected from the description section, and before the examples. Molecule images highlighted in blue are correctly annotated in PatCID. Molecules' SMILES are indicated.

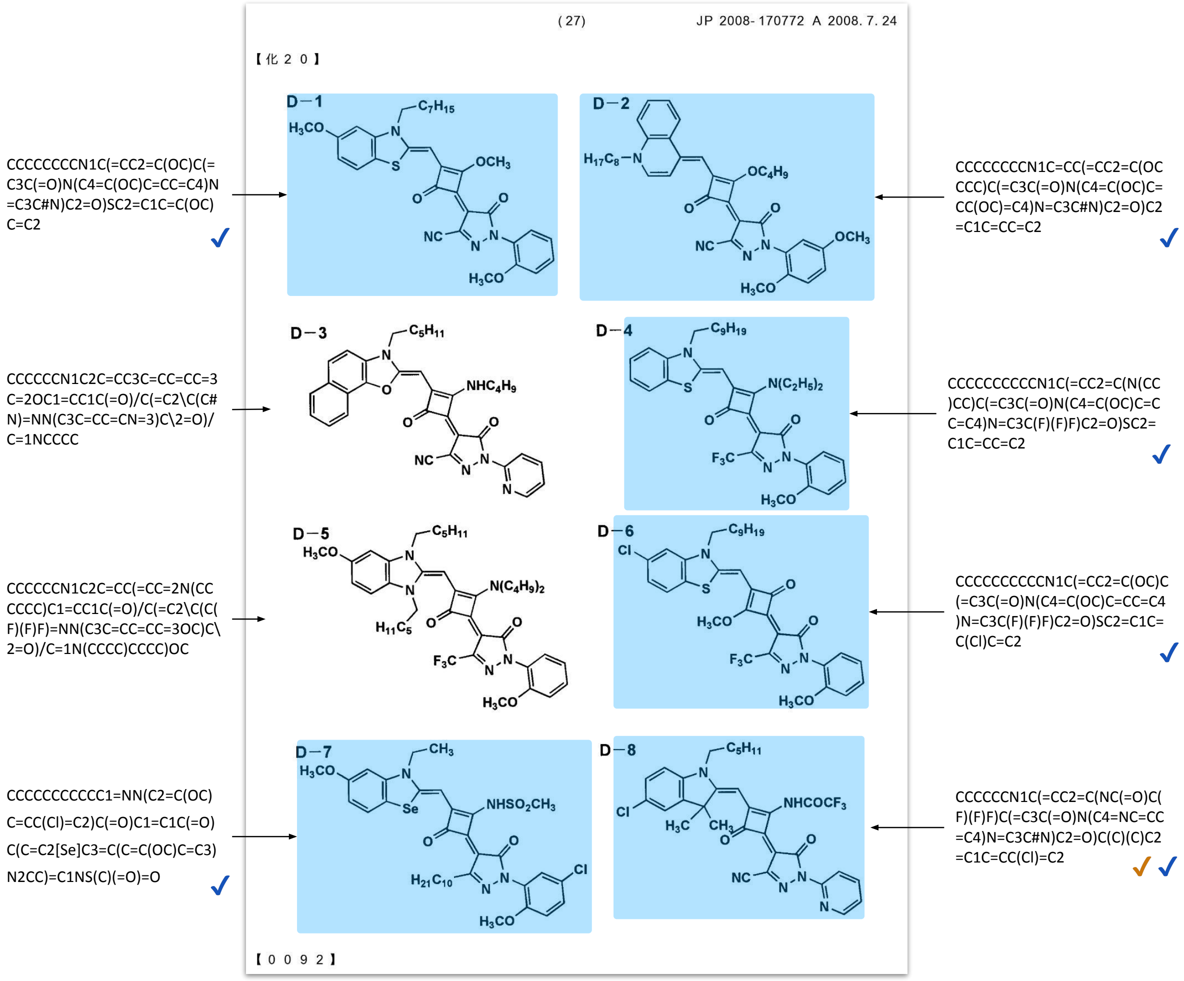

Supplementary Figure 4: **Qualitative example of molecules exclusively in PatCID.** Page from JP2008170772. SMILES with a blue check mark are found in PatCID. SMILES with an orange check mark are found in SciFinder.

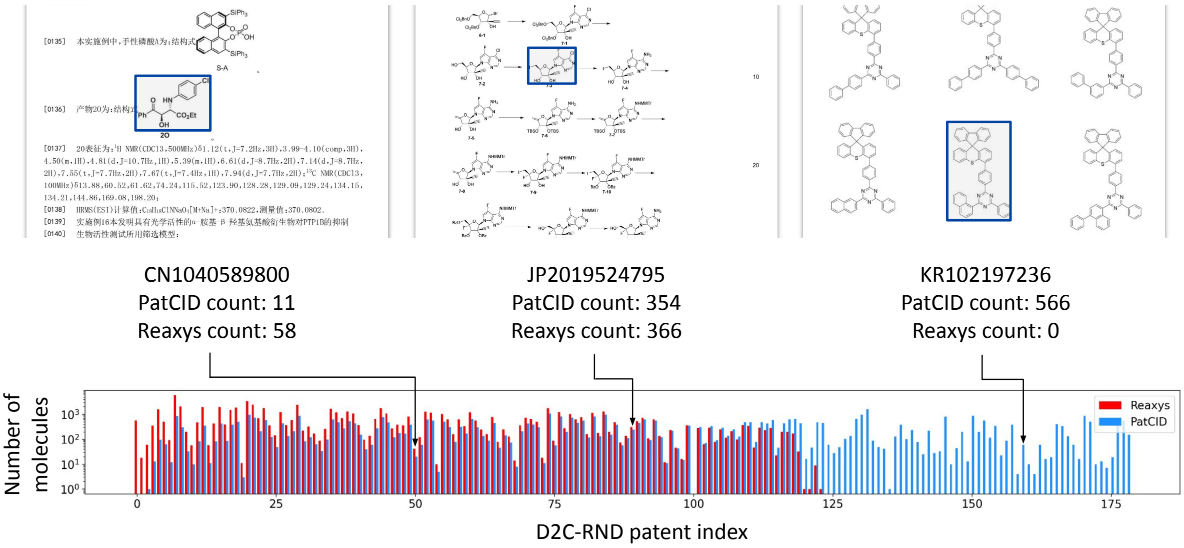

Supplementary Figure 5: **Molecule count comparison between PatCID and Reaxys.** Number of molecules annotated per patent in Reaxys (red) and PatCID (blue) for the random (D2C-RND) benchmark. Examples of reference pages of documents containing more molecules in PatCID (KR102197236), similar number of molecules in both databases (JP2019524795), and more molecules in Reaxys (CN1040589800). The patents are ordered with respect to the relative difference between the number of annotations in Reaxys and PatCID.

Supplementary Note 2

Analyzing the number of occurrences of molecules in PatCID can provide insights on the dataset quality. To begin with, it is expected that PatCID contains multiple occurrences of the same molecule. As illustrated in Supplementary Figure 6, the same molecule is generally displayed in all the patents from a family. Additionally, a molecule can eventually be displayed multiple times in the same patent, for instance, once as an example of embodiments, and a second time, as the product of a synthesis. As illustrated in Supplementary Figure 7, molecules with a very high number of occurrences are typically definitions of R-groups, reactants, solvents or counterions, which may not be of high value for searching information in patent documents. Supplementary Figure 8 shows the distribution of the number of occurrences of molecules in PatCID, counted once per document. 88% of molecules have less than 5 occurrences. It is consistent with the fact that PatCID contains documents from 5 patent authorities, and confirms that the vast majority of molecules in PatCID are not extracted from noise in patent documents. For the automatically-created database SureChEMBL [2], this analysis provides different results [3]. Extracting chemical structures from text leads to a larger number of irrelevant compounds. By only extracting molecule images, PatCID is able to filter most of this noisy information.

Besides, molecules that appear only once or twice in PatCID could be expected to be some errors from the recognition pipeline. Thus, we inspect 145 randomly selected images for which the predicted molecule appear only once in PatCID, and 86 for which the predicted molecule appears twice. We measure the percentage of correctly predicted molecules. In the first case, the precision is 45%, and in the second case, 73%. This is consistent with the evaluation on the D2C-RND benchmark (which gives a precision of 63%), and disproves the point that molecules with low number of occurrences are necessarily errors.

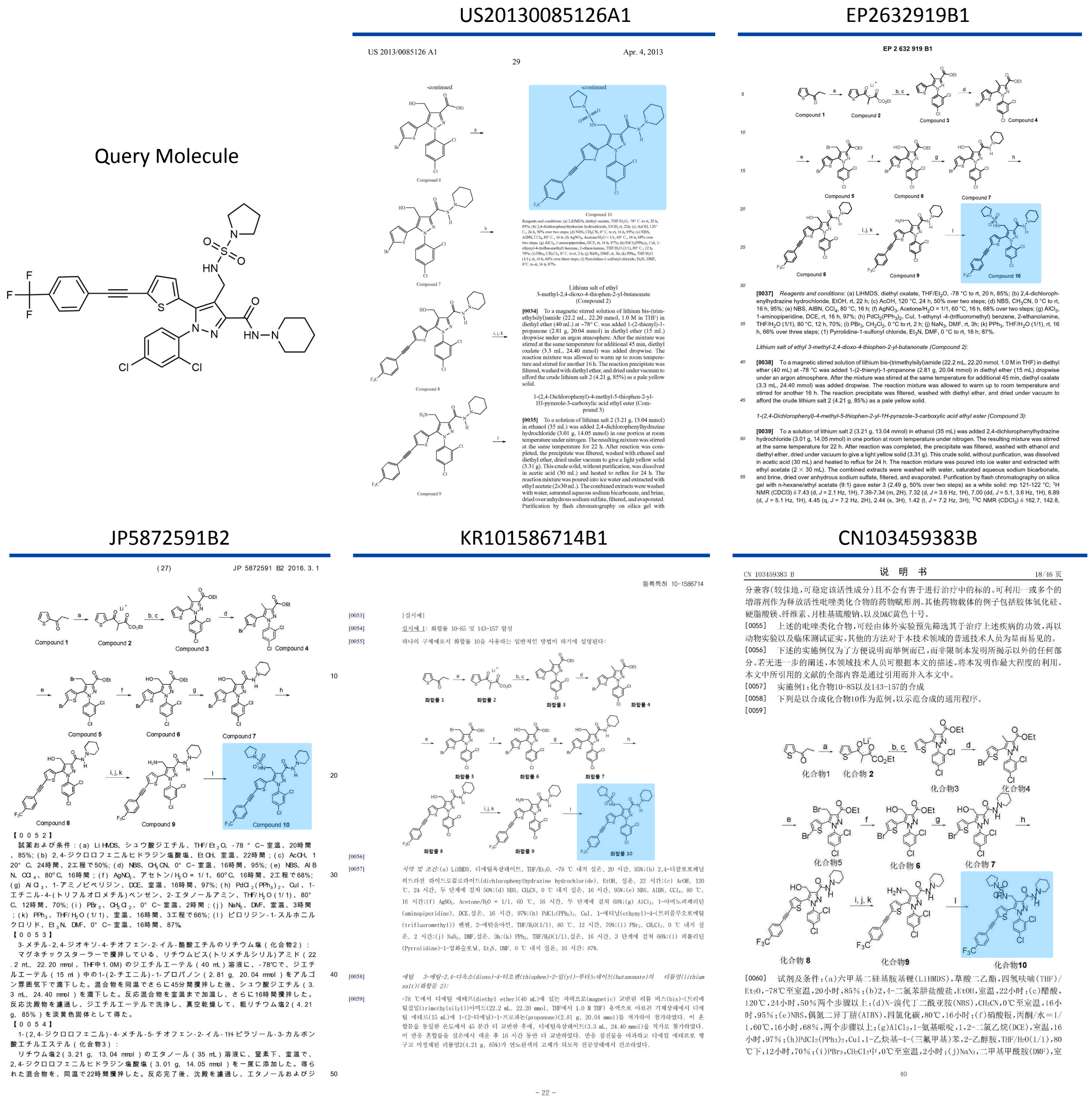

Supplementary Figure 6: **Same molecule displayed in patent family.** Example molecule found in 5 patent documents that disclose the same invention, published by different patent authorities.

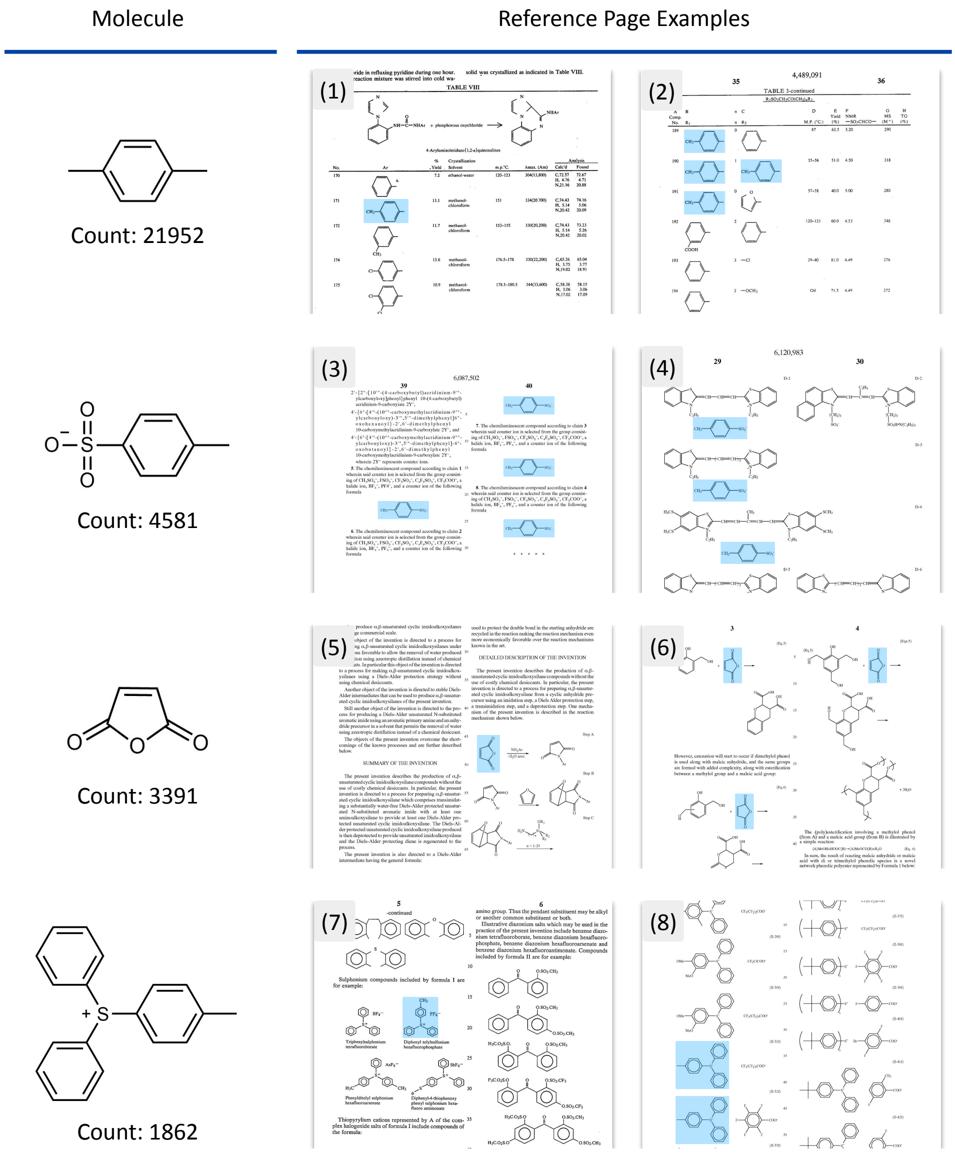

Supplementary Figure 7: **PatCID abundant molecules.** Example of molecules found with high occurrences in PatCID, with some of the pages from which the molecules were extracted. In the pages displayed, (1) and (2) are Markush structures R-group examples. (5) and (6) are reactants. (3), (4), (7) and (8) are counterions.

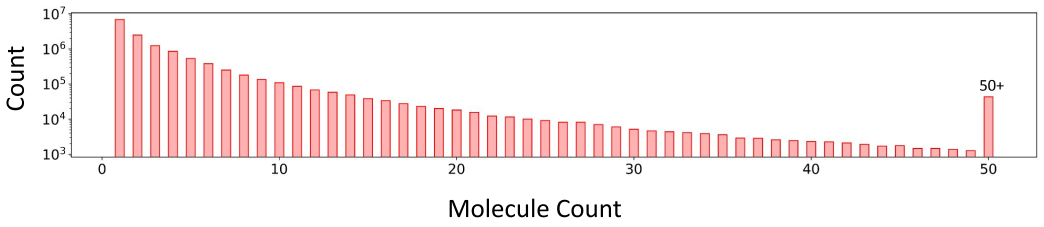

Supplementary Figure 8: **PatCID molecule count distribution.** The distribution of the number of occurrences of molecules in PatCID. Each molecule is counted once per document. The molecules flagged as error by MolGrapher are not included. All molecules with more than 50 occurrences are merged into the same bin. The first bar shows that almost 10M molecules are found only once in the PatCID database.

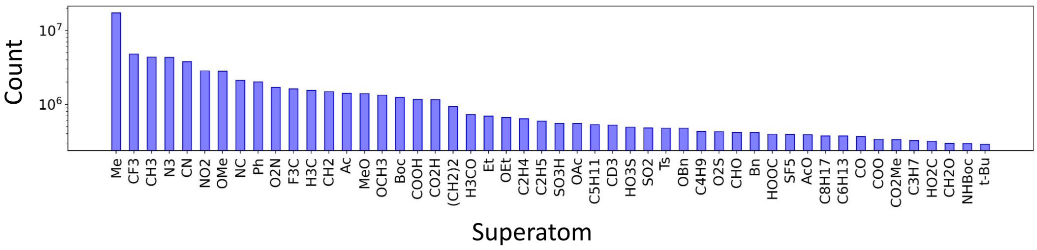

Supplementary Figure 9: **PatCID superatom distribution.** The number of occurrences of the 50 most common superatoms, i.e. abbreviated substructures, in PatCID. The superatoms are predicted by MolGrapher.

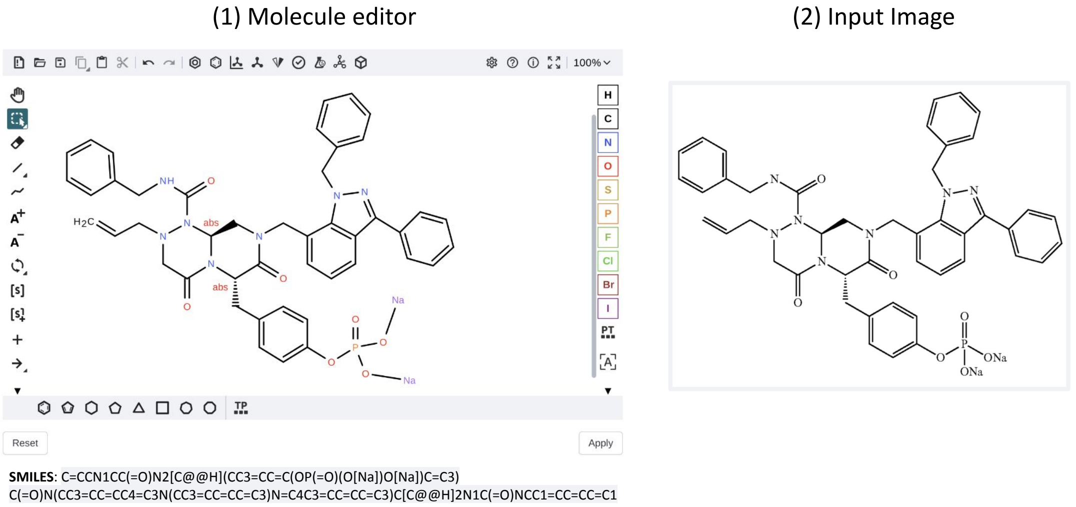

Supplementary Figure 10: **Chemical-structure annotation application.** The interface of the application built to annotate the chemical-structures images from benchmarks. Side-by-side visualization of the graph (1) and the molecular image (2) to be annotated.

Method	CPU	GPU	Number Parameters
DECIMER [4]	9.6 second/image	2.3 second/image	52M
MolGrapher [5]	4.6 second/image	0.5 second/image	39M

Supplementary Table 1: **Molecule recognition runtime comparison**. Runtime performance averaged over 100 input images selected from D2C-UNI. CPU refers to an inference with an Intel(R) Xeon(R) CPU E5-2690 v4 @ 2.60GHz, 16 threads and a batch size of 1. GPU refers to an inference with a Tesla V100-PCIE-16GB and a batch size of 1.

Databases	D2C-RND		D2C-UNI	
	Molecules (200)	Annotated Documents (179)	Molecules (164)	Annotated Documents (164)
**Text**				
SureChEMBL [2]	10.0%	45.3%	3.7%	15.9%
Google Patents	14.0%	41.3%	11.6%	48.8%
**Text and Image**				
SureChEMBL	23.5%	45.3%	6.1%	15.9%
Google Patents	41.5%	68.2%	17.7%	67.1%
**Image**				
SureChEMBL	22.0%	35.8%	4.9%	11.6%
Google Patents	36.5%	59.8%	9.8%	54.3%
PatCID	56.0%	100%	47.6%	98.2%

Supplementary Table 2: **Search comparison for textual or visual annotations.** Comparison of the molecule and document retrieval performances of patent databases, using textual or visual annotations. The recall of molecules and annotated documents is reported for benchmarks based on random (D2C-RND) and uniform (D2C-UNI) distributions of chemical images. The numbers in between parentheses are the numbers of samples in each set.

Supplementary References

[1] LexisNexis TotalPatent One. `https://www.totalpatentone.com` (Accessed: January 2024).

[2] Papadatos, G. *et al.* SureChEMBL: a large-scale, chemically annotated patent document database. *Nucleic Acids Research* **44**, D1220–D1228 (2015).

[3] Senger, S., Bartek, L., Papadatos, G. & Gaulton, A. Managing expectations: assessment of chemistry databases generated by automated extraction of chemical structures from patents. *Journal of Cheminformatics* **7**, 49 (2015).

[4] Rajan, K., Brinkhaus, H. O., Agea, M. I., Zielesny, A. & Steinbeck, C. DECIMER.ai: an open platform for automated optical chemical structure identification, segmentation and recognition in scientific publications. *Nature Communications* **14**, 5045 (2023).

[5] Morin, L. *et al.* MolGrapher: Graph-based Visual Recognition of Chemical Structures. In *Proceedings of the IEEE/CVF International Conference on Computer Vision (ICCV)*, 19552–19561 (2023).
